# Time-Limited Eating and Continuous Glucose Monitoring in Adolescents with Obesity: A Pilot Study

**DOI:** 10.3390/nu13113697

**Published:** 2021-10-21

**Authors:** Alaina P. Vidmar, Monica Naguib, Jennifer K. Raymond, Sarah Jeanne Salvy, Elizabeth Hegedus, Choo Phei Wee, Michael I. Goran

**Affiliations:** 1 Center for Endocrinology, Diabetes and Metabolism, Diabetes & Obesity Program, Department of Pediatrics, Children’s Hospital Los Angeles, Los Angeles, CA 90027, USA; mnaguib@chla.usc.edu (M.N.); jraymond@chla.usc.edu (J.K.R.); ehegedus@chla.usc.edu (E.H.); goran@usc.edu (M.I.G.); 2 Department of Pediatrics, Keck School of Medicine, University of Southern California, Los Angeles, CA 90027, USA; 3 Research Center for Health Equity, Cedars-Sinai Medical Center, Department of Medicine, Samuel Oschin Comprehensive Cancer Institute, Los Angeles, CA 90048, USA; Sarah.Salvy@cshs.org; 4 Department of Population and Public Health Sciences, Keck School of Medicine, Southern California Clinical and Translational Science Institute (SC-CTSI), Los Angeles, CA 90007, USA; cwee@usc.edu

**Keywords:** intermittent fasting, continuous glucose monitor, obesity, pediatrics, adolescents

## Abstract

Due to its simplicity, time-limited eating (TLE) may represent a more feasible approach for treating adolescents with obesity compared to other caloric restriction regimens. This pilot study examines the feasibility and safety of TLE combined with continuous glucose monitoring (CGM) in adolescents. Fifty adolescents with BMI ≥95th percentile were recruited to complete a 12-week study. All received standard nutritional counseling, wore a CGM daily, and were randomized to: (1) Prolonged eating window: 12 h eating/12 h fasting + blinded CGM; (2) TLE (8 h eating/16 h fasting, 5 days per week) + blinded CGM; (3) TLE + real-time CGM feedback. Recruitment, retention, and adherence were recorded as indicators of feasibility. Weight loss, dietary intake, physical activity, eating behaviors, and quality of life over the course of the intervention were explored as secondary outcomes. Forty-five participants completed the study (16.4 ± 1.3 years, 64% female, 49% Hispanic, 75% public insurance). There was high adherence to prescribed eating windows (TLE 5.2 d/wk [SD 1.1]; control 6.1 d/wk [SD 1.4]) and daily CGM wear (5.85 d/wk [SD 4.8]). Most of the adolescents (90%) assigned to TLE reported that limiting their eating window and wearing a CGM was feasible without negative impact on daily functioning or adverse events. There were no between-group difference in terms of weight loss, energy intake, quality of life, physical activity, or eating behaviors. TLE combined with CGM appears feasible and safe among adolescents with obesity. Further investigation in larger samples, with a longer intervention duration and follow-up assessments are needed.

## 1. Introduction

In the United States, one in five adolescents has obesity, and 30–50% of those go on to develop early onset type 2 diabetes, which is associated with a high risk of complications [1,2,3,4]. With increasing prevalence, an aggressive disease phenotype with risk for both short- and long-term health complications, and increasing cost for care, pediatric obesity in adolescents is expected to result in extensive financial costs, significant life-limiting complications, and negative impacts on quality of life [5,6,7,8]. Conventional pediatric obesity treatment addresses nutritional, physical activity, and behavioral topics with the goal of achieving clinically meaningful weight loss, defined as a weight loss of 5% or more of baseline weight [4,5,9,10,11,12,13,14]. Adherence to comprehensive lifestyle intervention recommendations is challenging for adolescents, in part because these approaches require monitoring and engaging in multiple behavioral targets (e.g., caloric intake and/or macronutrients, physical activity, impulse control). There is increased interest in finding effective and sustainable alternatives to improve weight loss and overall health and well-being in adolescence.

Multiple trials, conducted globally, in adult populations have examined the efficacy of various fasting regimens, including alternate day fasting, fasting mimicking diet, and time-restricted eating (TRE) [15,16,17,18,19,20,21,22,23,24,25,26,27,28,29,30,31]. Time-restricted eating involves shortening the eating window to a pre-specified number of hours per day (6 to 10 h) and fasting for the remaining hours of the day, without altering diet quality and quantity [30,31]. TRE has been shown to be well-tolerated and safe in adult populations, while promoting β cell responsiveness and reduction in fat mass [20,21,24,25,32,33,34,35]. However, the feasibility and effectiveness of TRE in adolescents has been questioned due to concerns of poor adherence, fear of iatrogenic adverse events (such as increased disordered eating behaviors [33,34,35,36]), and consequences on development. Because of its simplicity, TRE may result in greater intervention adherence than comprehensive and costly approaches, while preserving autonomy and dietary preferences [35].

This pilot study was undertaken to examine the feasibility and safety of TRE or time-limited eating (TLE, as it will be referred to moving forward) combined with a continuous glucose monitor (CGM) relative to eating during an extended eating window among adolescents with obesity. We were primarily interested in the feasibility of recruiting and retaining adolescents in the study, and examining adherence to the intervention and assessment procedures, while monitoring possible iatrogenic effects of TLE on eating attitudes and practices. CGM was used to capture glycemic excursions, monitor adherence to TLE and control intervention protocols, and monitor for hypoglycemia. Weight loss, dietary intake, percent time in range, quality of life, physical activity, and eating behaviors and attitudes were collected as secondary outcomes. We hypothesized that TLE would be feasible, safe, and not negatively impact any of the secondary outcomes during the 12-week trial. 

## 2. Materials and Methods

### 2.1. Study Design

This 12-week pilot randomized controlled trial examined the feasibility, safety, and preliminary efficacy of TLE (8-h eating/16-h fasting, intervention) compared to the control (12-h eating/12-h fasting). The trial was implemented remotely between March 2020 and June 2021 [36]. The protocol was reported by Vidmar et al.; however, due to the timing of implementation, there were several protocol changes made before implementation. Briefly, adolescents (ages 14–18) with obesity (BMI ≥ 95th percentile) were recruited from clinical programs at Children’s Hospital Los Angeles (CHLA). All participants and their families received a one-time, two-hour nutritional counseling session promoting low added sugar and carbohydrate intake delivered by a healthy educator. Participants chose and paid for their own food for the entire intervention. Research visits were conducted at 0, 4, 8, and 12 weeks and lasted 120 min (5 total visits, including initial consent visit). All participants wore a continuous glucose monitor (CGM) daily for the study duration. After a one-week run-in period, all participants were randomized (block size = 3 and 6 and balanced by sex and age) to: (1) Control: 12 h eating/12 h fasting + blinded CGM; (2) TLE (8 h eating/16 h fasting 5 days per week) + blinded CGM; and (3) TLE + real-time CGM feedback.

Due to COVID-19 restrictions, this pilot study was conducted virtually. Study material (body scales and CGM supplies) were shipped to the participants’ homes, and all study interactions with participants, including the informed consent process and enrollment into the study, occurred via a secure HIPPA-compliant videoconference platform. Experienced staff guided the participants to conduct anthropometric measurements throughout the study period. Participants completed validated patient-reported outcome surveys at each visit via Research Electronic Data Capture (REDcap). Weekly contacts with participants were conducted over the phone by the study team, lasting approximately 15 min per session. The purpose of these calls was to review participants’ experience with the prescribed eating window, provide support and guidance, and monitor for adverse events. Participants were also asked to report any adverse events or changes in their health or physical function since the last contact. See the full study protocol for details on study team training and fidelity monitoring [37].

All study procedures were approved by the CHLA Institutional Review Board (CHLA-000193, date of approval—20 December 2019). The study was reported according to the Consolidated Standards of Reporting Trials (CONSORT) statement and is registered with ClinicalTrials.gov (NCT03954223). Written informed consent was obtained from the adolescents and one parent or guardian. The study was conducted in accordance with the Declaration of Helsinki and all participants provided written informed consent prior to participation. Participants received compensation in the form of gift cards to complete study assessments.

### 2.2. Participants

Inclusion criteria were: (1) age 14–18 years; (2) BMI ≥ 95th percentile; (2) participant and/or parent/guardian or family member had a personal smart phone that was CGM compatible and/or was willing to come to the study center for manual data upload monthly for the study duration; and (3) participant was willing and able to adhere to the assessments, visit schedules, and eating/fasting periods Adolescents were ineligible for the study if they: (1) had a documented diagnosis of Prader Willi Syndrome, type 2 diabetes, brain tumor, hypothalamic obesity, binge eating disorder, serious developmental or intellectual disability, or previously diagnosed eating disorder; (2) were unable or unwilling to complete study assessments (e.g., inability to wear CGM, inability to be in the imaging modality without sedation); and/or (3) were enrolled in a weight loss intervention or previously underwent bariatric surgery; or (4) were taking weight-altering medication (e.g., antipsychotics, sedatives, hypnotics, off-label obesity medication, insulin).

### 2.3. Intervention Components

Components Common to All Study Arms. All participants received two hours of nutrition counseling focusing on reduction of carbohydrate and added sugar intake prior to randomization. The education session provided dietary recommendations for intake of added sugars (<5% of daily energy intake) and carbohydrate (<100 g per day). No specific caloric restriction was recommended, and participants were not required to keep logs of food intake. Recommendations were made in terms of avoiding sugar-sweetened beverages, juices, and food high in added sugars. In addition, physical activity consistent with physical activity guidelines for adolescents was encouraged but not formally prescribed.

Time Limited Eating. Participants were instructed to consume all their food in an eight-hour time window (i.e., from 11 AM to 7 PM) with a 16-h fasting period. Participants selected their eating window based on feasibility and daily routine. At baseline, participants’ eating windows were recorded based on dietary recall. At the consent visit, baseline eating windows were re-assessed, and participants were required to select their eating/fasting windows. Noncaloric, non-artificially sweetened beverages (water, tea, coffee) were allowed during the fasting period. All participants were asked to record the time they started and finished eating daily, and to report their eating windows with the study staff weekly.

Control. Participants assigned to the control arm were instructed to consume food over a 12-h or more eating window. No energy restriction was required. All participants were asked to record the time they started and finished eating daily and to report their eating windows to the study staff weekly. As described above, the nutrition and physical activity recommendations were received by all participants regardless of treatment group.

Continuous Glucose Monitor. All participants wore Dexcom G6 continuous glucose monitors (Dexcom, San Diego, CA, USA) continuously for 13 weeks (week −1 to week 12 of the study period). All participants were blinded to CGM data for seven days for baseline data collection (1 week), and then randomized to one of three intervention arms: Control + blinded CGM; (2) TLE + blinded CGM; and (3) TLE + real-time CGM feedback. Participants in the control and TLE + blinded CGM groups were blinded to the CGM data in that they did not have access to the smartphone app or web browser platform that housed the glycemic data, throughout the study period and therefore did not have real-time access to their glycemic profiles. Participants in the TLE + real-time CGM feedback group were coached to use a personal smart phone with Bluetooth capabilities to access real-time blood glucose levels throughout the 12-week intervention period. Participants were provided with a transmitter and enough sensors to replace the sensor every 10 days. The participants and guardians were educated on how to use the CGM and received 1:1 coaching on how to change the sensor, which was completed either independently or under study team guidance. No glucometer calibration was required. At each weekly phone meeting, study staff monitored any adverse events and challenges related to CGM wear, including participant discomfort, skin adherence, and other issues.

### 2.4. Measurements

At baseline, adolescents and their parents were asked to complete a demographic questionnaire (age, gender, race/ethnicity, household composition, education, household income), baseline eating window (assessed with dietary recall and semi-structured interview), and medical history. The primary endpoint of the study was feasibility. Feasibility was determined by assessing the number of days adolescents complied with their prescribed eating window, number of days they wore their CGM, number of weekly phone calls and scheduled research visits they attended, Satisfaction Questionnaire, and exit interview. Secondary goals for the study were to compare clinical outcomes (weight loss, dietary intake and quality, physical activity, eating behaviors and practices, and quality of life) for adolescents in the TLE versus control groups throughout the study period. Participants were also asked to complete a series of self-reported survey measures at baseline, mid-study, and three months. Measures included the Nutrient Data System Recall (NDSR) 24 Hour Dietary Recall, Pediatric Quality of Life Scale (PedsQL), Patient Reported Outcomes Measurement Information System (PROMIS^®^) Physical Activity Scale, and Binge Eating Disorder Screener (BEDS) [35,38,39,40,41,42,43]. Exploratory goals of the study were to compare glycemic profiles (percent time in range, average glucose) between TLE and control throughout the study period.

#### 2.4.1. Primary Outcome—Feasibility

Compliance with the recommended eating windows was collected from adolescents during the weekly phone calls with the study team. Adolescents were asked to record the time they started and finished eating daily, the number of days they adhered to their prescribed eating schedule, and barriers to adherence. Adolescents were instructed to wear their CGM daily for the duration of the study and to report deviation from the protocol during the phone calls. In addition, study staff reviewed the Dexcom Clarity platform to verify the number of CGM wear days per week. The number of calls completed over the course of the study was recorded. Assessment of satisfaction with the eating window included a 5-point scale from 1 = ‘strongly agree’ to 5 = ‘strongly disagree for the following domains: (1) perceived effects of eating window on daily functioning, (2) would recommend to friends, (3) perceived hunger, and (4) how the assigned eating window impacted their family. During weekly phone calls with the study staff, adolescents were asked open-ended questions about their experience with either TLE or control, likelihood of continuing their current eating window after the study was over, and any barriers to adherence. A one-time exit interview was completed at week 12.

#### 2.4.2. Secondary Outcome

Anthropometrics. All participants received a wireless Bluetooth scale upon consent. Participants’ height and weight were collected by the participant and parent/guardian at home with the research coordinator monitoring the measurement collection via a HIPAA compliant virtual platform. Height was measured using a portable wall height indicator tape ruler, accurate to 0.5 cm (Posh Rulers, Quick Medical, Issaquah, WA, USA). Weight was measured on a self-calibrating Etekcity Digital Body Weight Scale, accurate to 0.2 kg (Etekcity, San Diego, CA, USA). Adolescents wore minimal clothing during the height and weight measurements. BMI was calculated as kilograms per meter squared and BMI z-score (zBMI) and excess percent of the 95th percentile (%BMI_p95_) was determined utilizing the CDC growth charts.

Dietary Intake [38,41,42,43]: Twenty-four-hour dietary recalls using the Nutrient Data System Recall (NDSR) 24 Hour Dietary were conducted in duplicates (one weekend day and one weekday in control and one TLE day and one non-TLE day for those in the TLE groups) at three timepoints throughout the study. The procedures used in the 1985–1986 United States Department of Agriculture Continuing Survey of Food Intakes of Individuals (USDA-CSFII) were followed, and all recalls were collected in a personal interview via the virtual platform using a standardized protocol based on the “multiple pass” method, which was developed and tested by the USDA for use in the 1994–1996 CSFII in an effort to limit the extent of under-reporting.

Pediatric Quality of Life Scale (PedsQL): Quality of life was measured utilizing the Pediatric Quality of Life Scale (PedsQL), which is a 10-item questionnaire designed to assess quality of life parameters for youth under 19 years of age [36,44,45,46,47]. The PedsQL is a brief, standardized, generic assessment instrument that systematically assesses patients’ and parents’ perceptions of health-related quality of life (HRQOL) in pediatric patients [44,46,48].

Physical Activity: Physical activity was assessed using the Patient Reported Outcomes Measurement Information System (PROMIS^®^) [39]. The PROMIS^®^ instruments were developed using rigorous qualitative and quantitative methods and standardized to a reference population. The PROMIS^®^ measures for children have been found to demonstrate feasibility, internal consistency, construct validity, and responsiveness to change in a clinical setting. The physical activity survey is a self-administered 7-day recall instrument developed to assess the general levels of physical activity of children and adolescents.

Binge Eating Disorder Screener (BEDS) [49,50,51,52]. Given that adolescents with obesity are at high risk of binge eating disorder (BED) symptoms, our goal was to screen participants at baseline to ensure appropriate referrals are made in a timely manner. In addition, we monitored for BED symptomatology as a safety metric throughout the study period. Binge Eating Disorder Screener (BEDS-7) is a brief, valid, patient-reported screening tool for use in primary care and general psychiatry settings to identify individuals most likely to have BED and to facilitate further evaluation or referral to specialists. It has been validated in youth aged 12–21 years.

#### 2.4.3. Continuous Glucose Monitoring

CGM data were downloaded weekly by the study team. CGM data were evaluated continuously over the study period. This data was utilized to compute the following measures: mean, maximum, and minimum glucose levels; standard deviation of glucose; mean amplitude of glycemic excursion; and overall percent of total time spent in euglycemic range (percent time in range = 70–180 mg/dL). All CGM data were housed in Clarity (Dexcom and Dexcom CLARITY are registered trademarks of Dexcom, Inc., San Diego, CA, USA) and the study team had weekly access to assess all glycemic excursions that occurred during the self-reported fasting periods [50,51,52,53]. For those in the TLE + real-time CGM feedback group, every time they viewed their CGM data in the app, the event was captured, and time stamped in the Clarity system.

### 2.5. Statistical Analysis

The study was a pilot trial, thus we opted for a convenient sample size of 50 participants to estimate parameters for a larger, fully powered trial. NDSR, PedsQL, PROMIS^®^ PA, and BEDS questionnaires were summarized according to prior literature. Analyses were based on the intention to treat (ITT) population using the last observation carried forward. The ITT population was defined as at least two visits (baseline and 1 month). The study was designed as a three-group intervention analysis; however, given that very few adolescents in the TLE+ real-time CGM feedback group looked at their real-time data, we completed a post hoc analysis combining both TLE groups compared to control for all analyses performed.

Baseline characteristics (age, sex, race, BMI status, household income) were summarized descriptively across arms for the ITT population using mean and standard deviation (SD) or median and interquartile range as appropriate for the distribution of continuous variables. Categorical variables are described as a frequency and percentage. Continuous variables that are skewed were analyzed in log scale. The differences in demographics, anthropometrics baseline, and eating window distributions among intervention groups were examined using analysis of variance (ANOVA) and Fisher’s Exact test. Adherence was operationalized as the number of days adolescents complied with their prescribed eating window, number of days they wore their CGM, and number of weekly phone calls and scheduled research visits they attended, and satisfaction scores were summarized using mean, standard deviation (SD), minimum, and maximum score between TLE and control.

To assess the TLE effect on the secondary outcomes, mixed-effects models were used to evaluate the change in clinical outcomes from week 0 to week 12 between intervention groups. The TLE effect on mean change of BMI z-score and %BMI_p95_ between week 0 and week 12 was assessed by using ANOVA. Then, a mixed-effects generalized linear model based on a Gaussian or Gamma distribution as appropriate was used to further assess the TLE effect on change in weight outcome from week 0 to week 12. The mixed-effects generalized linear model based on a Gaussian or Gamma distribution was used as appropriate for the distribution of continuous outcome variables. Whereas a mixed-effects logistic regression model was used for binary clinical outcome variables. In addition, a mixed-effects Tobit regression model was used to evaluate the TLE effect on the change in quality-of-life assessment, where the scores are reported in percentages, with no data below 0 or above 100. Then, the non-additive effects of TLE were also examined; specifically, whether the change on clinical outcomes during the study period varied by intervention groups by including the interaction term in the mixed-effect models. In addition, sensitivity analysis was performed to examine whether the weight change observed in the data was influenced by one adolescent who achieved a weight loss of greater than 15% from baseline weight. All results are described in beta estimate, β, percent change, and odds ratio with its associated 95% confidence interval and *p*-value. The statistical significance level was set at 0.05 with two-sided throughout the analyses. All statistical computations were done in Stata/SE 17.0 (StataCorp, College Station, TX, USA).

## 3. Results

### 3.1. Primary Outcome—Feasibility

#### 3.1.1. Characteristics of Participants Recruited

**Figure 1:**
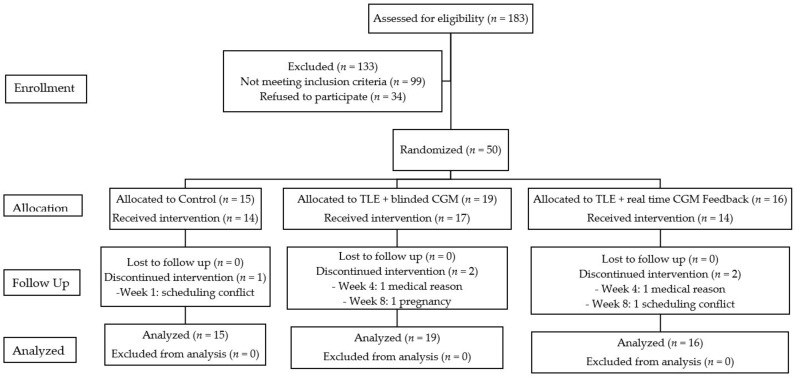
Consolidated Standards of Reporting Trials (CONSORT) flow diagram of participant inclusions.

Descriptive statistics are provided in Table 1 describing participants’ demographic characteristics and anthropometrics at week 0. In total, 511 adolescents with obesity were screened (Figure 1). Eligible adolescents were identified through various recruitment methods including clinic referral, hospital-wide advertising, community advertising, social media, and direct contact from the research team either by phone, email, or self-referral. Of the 183 adolescents screened, 99 did not meet the eligibility criteria. Of the remaining 84, 34 declined to participate. Thus, 50 adolescents were enrolled in the study, which achieved a recruitment rate of 60% of those who were contacted about the study. Five participants withdrew, two of whom developed type 2 diabetes and required initiation of pharmacotherapies, two withdrew because of unexpected changes to their school and work schedules, and one became pregnant. Forty-five adolescents completed the study (Figure 1). Consistent with the demographics of patients served by CHLA, most participants were Hispanic (60%), publicly insured (74%), and had an annual household income <$50,000 (70%) (Table 1). There was no significant difference in demographics or baseline characteristics between study completers and non-completers (all *p*-values > 0.5).

#### 3.1.2. Adherence

The baseline average eating period for this cohort was 9.7 h per day (SD 3.3, range: 1–21 h) with no significant difference between an eating window <10 h and ≥10 h among the three intervention groups (χ^2^ = 2.4, *p* = 0.3). Ninety percent of adolescents in the TLE groups selected to start their eating window between 10 AM and 12 PM (11 AM-7 PM—16/31, 52%, 12 PM and 8 PM—9/31, 29%), and 10 AM and 6 PM 3/31, 10%). The remaining three adolescents selected 1 PM-9 PM, 2 PM-10 PM, and 3 PM-11 PM. Eighty percent of adolescents in the control group selected to start their eating window between 10 AM and 1 PM and to end their eating window between 9 PM and 11 PM. Over the course of the 12-week study period, there was a significance difference in eating windows between TLE (TLE + blinded CGM and real-time CGM feedback: 7.0 h, SD 2.3) and control (9.8 h ± 2, SD 3.1, *p* < 0.001). Overall, adolescents were highly adherent to the prescribed eating periods (mean number of days in which TLE was completed per week: 5.2 d/week (SD 1.1) and mean number of days in which control was completed per week: 6.1 d/week (SD 1.2), Table 2). To better characterize adolescents’ eating windows, we compared self-reported fasting periods with CGM data to classify what glycemic excursions occurred during fasting. During fasting periods, only four adolescents had excursions ≥60 mg/dL, two of them being later diagnosed with T2D. The remaining participants had excursions from 0–99 mg/dL with a mean excursion of 65 mg/dL during fasting periods.

Based on adolescents’ responses to the satisfaction survey and exit interviews, TLE was viewed favorably. Overall, 90% of adolescents reported that the study was worthwhile and 95% reported that they would recommend it to others. Only 15% of adolescents reported barriers to implementing their assigned eating window into their daily schedule including conflict with work or sleep schedule, social commitments, and explaining eating patterns to family. Adolescents denied any negative compensatory behaviors (i.e., excessive exercising, binge episodes, or excessive dietary restraint). Adolescents in the TLE groups reported that eating within an 8-h daily period would be feasible for most adolescents and that they would recommend it to their peers. All participants reported they would be willing to continue to eat during their assigned eating window after the study was completed. When asked how helpful TLE was, on a scale from 1 (not helpful) to 5 (very helpful), the mean score was 4. Similarly, when asked how enjoyable the study was on a scale of 1 (not enjoyable) to 5 (very enjoyable), the mean score was 4, with no difference between groups. In addition, adolescents reported favorable experiences with wearing a CGM daily for 12 weeks. Adolescents wore their CGM for a mean of 5.85 (4.08 SD, median 7 days) days per week over the study period with no difference between groups (*p* = 0.9). No significant barriers to wearing the CGM daily were identified. One-third of participants (15/50) reported at least one minor barrier to daily CGM (i.e., skin irritation, mild bleeding at insertion site, etc.).

### 3.2. Secondary Outcomes

#### 3.2.1. Weight Loss

**Figure 2:**
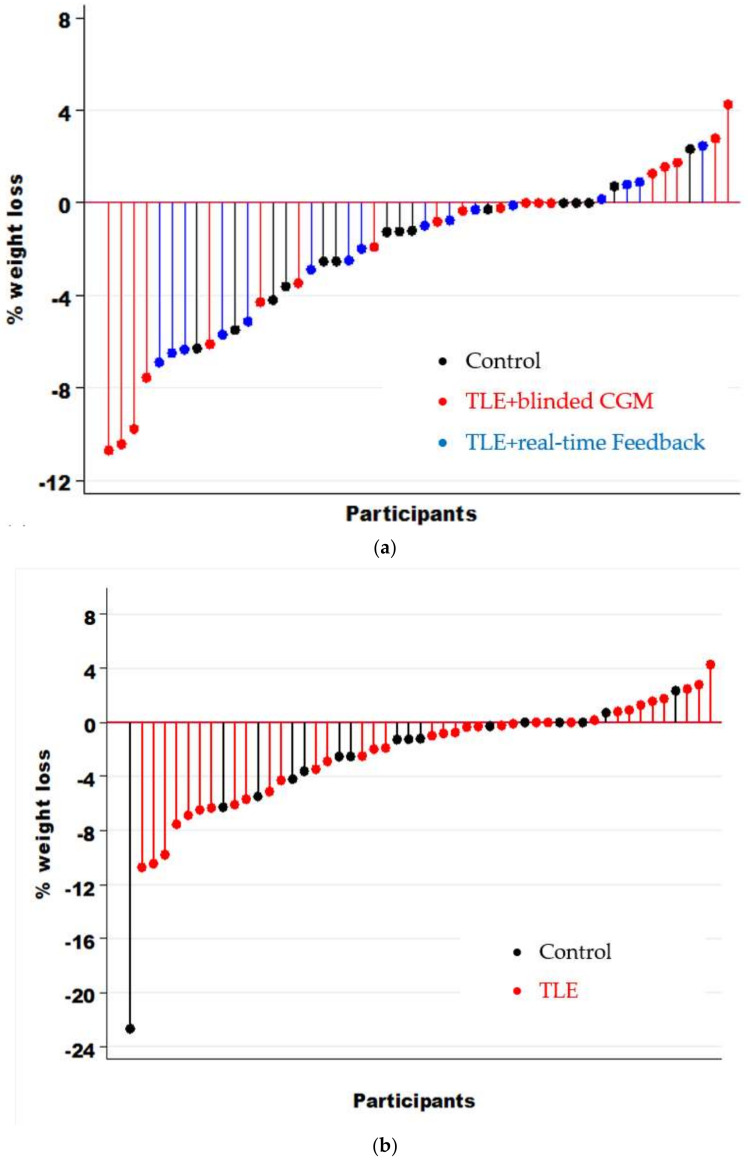
Percent weight loss at week 12 compared to baseline by individual participants across: (**a**) all three intervention arms and (**b**) TLE groups (TLE + blinded CGM and TLE + real-time CGM) compared to control.

**Figure 3:**
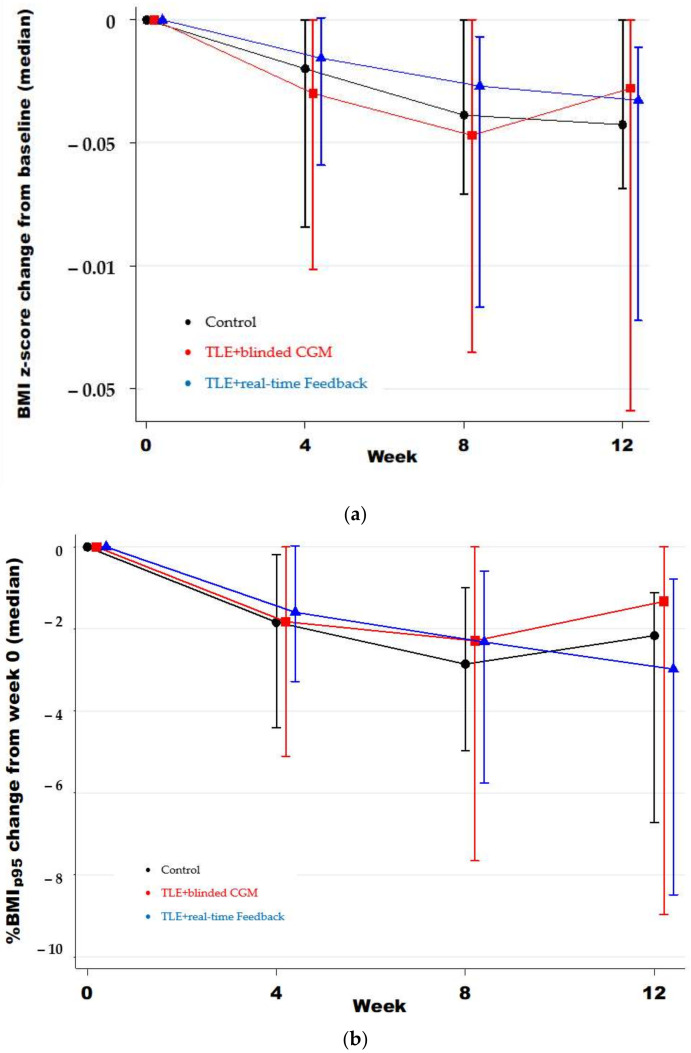
Weight change across the study period by intervention group for (**a**) change in excess percent of the 95th percentile (%BMI_p95_) and (**b**) change in BMI z-score.

There was great heterogeneity in weight loss across participants. Overall, 68% of adolescents lost weight during the intervention period (Figure 2). Post-intervention, 26% of the TLE + blinded CGM group lost ≥5% of their baseline weight vs. 31% in the TLE + real-time CGM feedback and 13% in the control group (no between-group difference *p* = 0.5). Consistent with intention-to-treat analysis, across the study period, there was a significant decrease in median weight loss (kg), %BMI_p95_, and zBMI across all three groups, with no significant difference in weight loss between groups from the mixed-effect generalized linear models (Table 3 and Table 4, and Figure 3). Sensitivity analysis was conducted to exclude one participant in the control group that lost >15% of their total body weight and the results remained the same. In addition, given that very few adolescents in the TLE+ real-time CGM feedback group looked at their real-time data, we completed a post hoc analysis combining both TLE groups compared to the control and the results remained the same (all *p* > 0.05).

#### 3.2.2. Dietary Intake and Quality

Overall, all adolescents showed a 25% reduction (~375 calories/day) in their total daily caloric intake on both weekdays and weekend days, and TLE and non-TLE days for those in the TLE groups (all *p* < 0.05). There was no difference in caloric reduction between groups (all *p* > 0.05). There was a small but significant reduction in the percent of calories consumed from added sugars (−2%, decrease of ~14.5 g/day) and carbohydrates (−5%, decrease of ~50 g/day) at week 12 compared to baseline across all participants, with no significant difference between groups.

#### 3.2.3. Physical Activity

All participants reported an increase in the number of days per week they participated in physical activity across the study period (mean +1 day/week (Range: −1 to 3 days/week). The mean t-score on the PROMIS^®^ Physical Activity questionnaire increased over the study period by 3.12 points (*p* = 0.01, minimally important clinical difference on PROMIS^®^ Physical Activity is 2–3 points) across all participants, with no significant difference between intervention arms (*p* = 0.7).

#### 3.2.4. Eating Behaviors and Attitudes

At baseline, 25% of adolescents reported some excessive eating behaviors, with 18% of them reporting distress from excessive eating. Participants who endorsed excessive eating at baseline were referred to their primary care provider for evaluation, and referral to a psychiatrist. Of those 25% identified at baseline, upon further evaluation by a psychiatrist, none met criteria for an eating disorder based on the DSM V criteria. The mixed-effect logistic regression model showed there was no significant change in excessive eating behaviors, binge eating episodes, or distress related to eating over the study course within and between groups (control vs. TLE—OR 3.1, 95% CI 0.64, 17.89, *p* = 0.1). During the exit interviews, none of the adolescents reported unhealthy eating behaviors, such as excessive exercise, dietary restraint, or eating disorder symptomatology, upon completion of the study.

#### 3.2.5. Quality of Life

The overall mean summary score on the PEDsQL scale significantly increased over the study duration (~10% increase, *p* < 0.01), with no significant difference noted between groups (*p* = 0.7. The mean summary (adolescent—β: 3.29, 95% CI: (1.12, 5.46), *p* = 0.003), psychosocial (adolescent—β: 4.50, 95% CI: (1.79, 7.21), *p* = 0.001), and physical health scores (β: 1.39, 95% CI: (−1.50, 4.29), *p* = 0.3) across all three groups significantly increased over time, with no significant difference between groups (all *p* > 0.05). There was no significant change in the mean summary score at week 12 compared to baseline depending on the intervention group (interaction *p* = 0.8). There was no significant effect of eating window (TLE vs. control) or blinded vs. unblinded CGM group (blinded CGM vs. real-time CGM feedback) on the summary score (all *p*-values > 0.5). On the mixed logistic regression model, there was no association between weight loss and improvement in the mean summary score (95% CI: −0.02, 30.99, *p* = 0.2)

### 3.3. Continuous Glucose Monitoring

There was no serious hypoglycemia reported in this cohort. There was no difference in the rates of hypoglycemia between groups (control as reference—TLE + blinded CGM: β = 1.3, 95% CI: (−1.5, 4.2), *p* = 0.3 and TLE + real-time CGM: β = 1.4, 95% CI: (−9.0, 6.1), *p* = 0.4). We evaluated whether adolescents randomized to the TLE + real-time CGM feedback engaged with the glycemic data during the study duration. Although all participants in this group had access to their glycemic data, only nine of the adolescents opened the application to review their glycemic data during the study. Post hoc analysis was completed, and there were no statistically significant differences in weight loss between those who did access the GCM data and those who did not (all *p* > 0.05). There was no difference in the reduction of average glucose levels or percent time in the range between TLE and control (all *p* > 0.5, Table 5).

## 4. Discussion

This is the first study to investigate the feasibility, safety, and preliminary efficacy of TLE in adolescents with obesity. We were able to recruit and retain adolescents in the study, and most participants were able to adhere to the prescribed eating windows. In addition, adolescents reported that TLE was a feasible approach, and it did not interfere with their normal daily patterns and social engagements. Like previous longitudinal monitoring of eating patterns in adults, the eating times in this group at baseline varied considerably [54,55]. Eating events were spread over a wide period of the day for many adolescents (1–24 h). Most participants selected an afternoon/evening eating window regardless of the assignment to the control or TLE groups.

Although we found no between-group difference in weight change, one-third of adolescents in the TLE groups and one-quarter of the control group achieved clinically meaningful weight loss of more than 5% of their baseline weight. One possible explanation for the absence of between-group difference lies in the structured day hypothesis [56,57,58]. Structured eating has been shown to produce weight loss in adult and pediatric populations [55], and all study participants were provided with a prescribed eating window (i.e., 8-h vs. 12-h eating window). Conceivably, adherence to a controlled eating schedule may help explain weight loss in a subset of adolescents in both study arms [55,59,60,61], especially considering the unprecedented disruptions created by the COVID-19 pandemic on adolescents’ schedules and daily activities [62,63,64,65,66].

The absence of a between-group difference may also be due to the eating window selected by participants assigned to TLE. All adolescents assigned to TLE selected an afternoon eating window. This finding aligns with a previous study done by this group, which showed that the majority of adolescents with obesity prefer an afternoon/evening eating window [67]. Available evidence in animals and humans suggests that early TLE (i.e., tantamount to skipping the evening meal) is more effective than late TLE (i.e., equivalent to skipping breakfast) for weight loss and metabolic benefits [28,56,57,68,69]. These findings have been explained in terms of alignment between central and peripheral circadian clocks involved in energy expenditure and fat oxidation [28,56,57,68,69]. In the present study, we allowed adolescents to select their own eating window to promote compliance, resulting in a late TLE regimen. Studies are needed to examine the feasibility of early TLE in adolescence and to compare the effectiveness of early and late TLE in adolescent and adult populations.

An alternative explanation for the absence of a difference in weight loss across study arms lies in the possible interventional effect of CGM. It is well-documented that wearable technology often results in a short-term weight loss; however, reactive effects are usually short-lived [58,70]. Only one-third of adolescents in the real-time CGM group looked at their data; however, participants’ mere knowledge that their glucose was monitored by the study team may have provided accountability, not provided outside the study. Additional work is needed to explore the role of CGM, with and without real-time biofeedback, in dietary intervention trials.

Akin to findings reported in adult cohorts, the assigned eating window (TLE vs. control) did not adversely affect quality of life, physical activity, or eating behaviors [71,72,73]. In this sample of adolescences, TLE was associated with a modest improvement in quality of life relative to baseline, with no difference compared to the control [73]. It has been widely reported that weight loss has a positive impact on quality-of-life measures after short-term interventions [74,75]; however, improvement in self-reported quality of life was not related to weight loss in the present study. Compared to a prolonged eating window, TLE did not impair physical activity. Interestingly, all adolescents showed an increase in the number of days of physical activity per week over the course of the study. These findings contrast the many reports documenting decreased physical activity during the COVID-19 pandemic [62,64,65,76,77], although not entirely surprising as children were not held to classroom schedules involving long periods of sedentary time [63,65,78].

TLE did not result in any unhealthy compensatory eating behaviors [71,72,79,80,81]. This finding is important given the concerns that TLE may lead to unhealthy eating behaviors and attitudes. Disordered eating behaviors are prevalent among adolescents with obesity; however, many studies have suggested that monitored intervention programs implemented by trained professionals may decrease eating behavior symptomatology [82,83,84]. The potential for unhealthy eating should be continuously monitored in future studies [73,83,85].

In most adult trials, TLE inadvertently reduced daily caloric intake and thus lead to weight loss [26,29,30,31,63,86,87]. Despite no recommendations to decrease caloric intake, there was a 25% reduction in daily caloric intake during the intervention compared to baseline with no difference between groups. As outlined above, this was likely secondary to increased daily eating structure and the consistency of the eating window. Additionally, although there was a wide variability of eating patterns at baseline, most adolescents were eating late into the evening and night and this night-time eating was limited with a structured eating pattern and may have contributed to caloric reduction [73,88]. It remains unknown if limiting night-time eating impacts caloric intake in adolescents; however, a large longitudinal study in adults with obesity comparing early and late eating periods found that timing of meal consumption was not associated with decreased caloric intake [73,88]. Additional investigation is needed to determine if TLE in adolescents is associated with consistent caloric reduction independent of the timing of the eating window.

Finally, as an exploratory outcome, we examined CGM use in this cohort as both a metric of adherence and an intervention modality. The efficacy of TLE interventions is dependent on accurate assessment of eating vs. fasting windows and therefore in this sample, CGM proved to be a useful tool to monitor the effect of fasting on glycemic profiles. By adding CGM data to self-report and dietary recalls, we were able to better understand the eating and fasting periods of this group and evaluate how the glycemic profiles changed during fasting. Certainly, this method is not without limitations in that glycemic excursions vary significantly based on the dietary macronutrient composition. However, the combination of dietary recall, self-report, and CGM data provides a strategy to evaluate adherence to fasting windows and useful data to inform future studies regarding expected glycemic excursions during fasting in adolescents with obesity without diabetes. No studies to date have investigated whether extended fasting periods increase risk of hypoglycemia in youth with obesity given their potential risk for glucose dysregulation. As a safety metric, we examined the frequency of hypoglycemia reported over time within and between groups in this cohort. We defined glucose based on the threshold of insulin secretion in the fasting condition in otherwise healthy adults as 70 mg/dL. Consistent with reports in adult cohorts without diabetes, there was no hypoglycemia noted during reported fasting periods, suggesting that prolonged fasting periods is not a risk factor for hypoglycemia in this age group. The CGM data also provided the opportunity to monitor average blood glucose and estimate HbA1c over time. Our findings are consistent with those previously report in adults in that we did not see a significant change in glucose regulation over the study period between groups because our cohort had baseline normal fasting glucose levels. In many adult trials, TLE has been associated with a reduction in fasting glucose and insulin sensitivity in participants whose baseline fasting glucose was >100 mg/dL [89,90]. The impact of TLE on glucose regulation may be related to severity of beta cell dysfunction at baseline and therefore further studies are needed in youth with pre-diabetes and type 2 diabetes to understand the impact of TLE on glucose regulation.

### Limitations

This study is not without limitations. First, given the COVID-19 research restrictions, our study was conducted entirely remotely. We were neither able to collect and verify all anthropometric outcomes nor collect body composition measures and other metabolic markers as initially intended, which added more variability to our analysis. Second, as this was a pilot study, we had a small sample size and were not powered to evaluate our secondary outcomes. Thirdly, the study was not conducted in a controlled or inpatient setting. We intentionally conducted our study in a real-life setting. We encountered unique barriers to recruitment, brought on by the pandemic, with evolving restrictions and unexpected delays during the research period. Fourthly, given this was a pilot trial, we did not exclude participants with a shorter eating window at baseline and required adolescents to adjust their eating window based on their randomization arm. In addition, our design is also subject to omitted variable bias, such as unmeasured or uncontrolled factors (i.e., impact of COVID-19 during the study period). Fifth, the current study could not evaluate whether TLE is sustainable over the long term given the short study duration. Although adherence was high for the study duration, further investigation is warranted to assess if TLE is more sustainable than other caloric restriction approaches given its simplicity and ability to be implemented in a real-life setting. Finally, our sample strictly included adolescents enrolled in a weight management intervention. Our focus on treatment-seeking adolescents is important to characterize the heterogeneity and specific needs of adolescents who seek obesity treatment.

## 5. Conclusions

Our results suggest that TLE, combined with CGM, is feasible, acceptable, safe, and can lead to clinically meaningful weight loss. All adolescents in the TLE groups selected an afternoon/evening eating window. TLE did not result in changes in physical activity, quality of life, or compensatory eating behaviors. Further research is needed to determine the effectiveness of TLE + CGM on weight reduction in larger cohorts, over longer intervention periods, and to investigate the optimal timing of TLE to produce the greatest weight reduction and improved health outcomes in this age group.

## Figures and Tables

**Table 1:** Demographic characteristics and baseline anthropometrics.

	Total (*n* = 50)	Control (*n* = 15)	TLE + Blinded CGM (*n* = 19)	TLE + Real-Time CGM Feedback (*n* = 16)	*p*
Age (in year) ^1^	16.43 ± 1.17	16.38 ± 1.25	16.16 ± 1.16	16.80 ± 1.09	0.3 ^a^
Sex ^2^					0.8 ^b^
Male	14 (28.0)	3 (20.0)	6 (31.5)	5 (31.2)	
Female	36 (72.0)	12 (80.0)	13 (68.4)	11 (68.7)	
Race ^2^					0.05 ^b^
White	5 (10.0)	3 (20.0)	1 (5.2)	1 (6.0)	
Black	3 (6.0)	1 (6.6)	2 (10.5)	0 (0)	
Asian	4 (8.0)	3 (20.0)	1 (5.2)	0 (0)	
Hispanic	27 (54.0)	7 (46.7)	13 (68.4)	7 (43.8)	
Am. Indian	2 (4.0)	0 (0)	1 (5.2)	1 (6.2)	
Mixed race	6 (12.0)	1 (6.6)	0 (0)	5 (31.2)	
Ethnicity ^2^					0.1 ^b^
Non-Hispanic	15 (30.0)	8 (53.3)	4 (21.1)	3 (18.7)	
Hispanic	32 (64.0)	7 (46.6)	14 (73.6)	11 (68.7)	
Weight (kg) ^3^	101.4 (87.9, 123.8)	104.3 (74.8, 123.1)	99.5 (84.6, 123.2)	110.5 (92.2, 128.3)	0.9 ^c^
%BMI_p95_^3^	125.9 (111, 158)	141.1 (114.4, 167.0)	122.6 (110.0, 158.5)	123.9 (109.8, 159.1)	0.9 ^c^
BMI z-score ^1^	2.30 ± 0.5	2.34 ± 0.5	2.28 ± 0.4	2.30 ± 0.5	0.9 ^a^

^a^ Analysis of variance; ^b^ Fisher’s Exact test; ^c^ Analysis of variance in log scale; ^1^ Mean ± standard deviation; ^2^ Frequency (percentage); ^3^ Median (interquartile range).

**Table 2:** Mean number of days (SD) in which the assigned eating window was completed across intervention arms using intention to treat with carry forward of the last weeks data.

Week	Control (*n* = 15)	TLE + Blinded CGM (*n* = 19)	TLE + Real-Time CMG Feedback (*n* = 16)
1	5.1 (1.9)	4.03 (1.9)	4.5 (1.9)
2	5.6 (1.3)	5.3 (1.3)	5.3 (1.2)
3	5.6 (1.3)	5.4 (1.3)	6.7 (1.3)
4	6.1 (1.6)	5.1 (1.6)	5.3 (1.4)
5	5.9 (1.6)	5.1 (1.6)	5.4 (1.7)
6	4.9 (1.3)	5.4 (1.3)	5.9 (1.1)
7	6.7 (1.3)	5.3 (1.3)	6.0 (1.0)
8	5.4 (1.1)	5.4 (1.1)	4.9 (1.1)
9	5.9 (1.0)	5.1 (1.0)	4.8 (1.5)
10	6.1 (1.2)	5.3 (1.2)	5.3 (1.4)
11	5.5 (1.5)	5.3 (1.5)	5.1 (1.0)
12	4.9 (1.0)	5.3 (1.0)	4.9 (1.1)

**Table 3:** Weight change between baseline and week 12 across intervention arms.

Weight Change	Control (*n* = 15)	TLE + Blinded CGM (*n* = 19)	TLE + Real-Time CGM Feedback (*n* = 16)	*p*	Effect Size
BMI z-score change	−0.05 ± 0.09	−0.09 ± 0.14	−0.11 ± 0.19	0.6	0.04
%BMI_p95_ change	−3.27 ± 3.34	−3.76 ± 5.76	−4.85 ± 5.08	0.7	0.04

**Table 4:** Gamma mixed-effects generalized linear model on %BMI_p95_ and BMI z-score.

%BMI_p95_	% Change	95% CI	*p*
**Week**			
0	Ref	--	--
4	−2.0	(−2.7, −1.3)	<0.0001
8	−2.9	(−3.8, −1.9)	<0.0001
12	−3.3	(−4.4, −2.1)	<0.0001
**Intervention group**			
Control	Ref	--	--
TLE + blinded CGM	−3.4	(−17.6, 13.3)	0.7
TLE + real-time CGM feedback	−4.3	(−17.9, 11.7)	0.6
**BMI z-score**	β	95% CI	*p*
**Week**			
0	Ref	--	--
4	−0.05	(−0.1, −0.03)	<0.0001
8	−0.08	(−0.1, −0.04)	<0.0001
12	−0.09	(−0.1, −0.05)	<0.0001
**Intervention group**			
Control	Ref	--	--
TLE + blinded CGM	−0.08	(−0.4, 0.2)	0.6
TLE + real-time CGM feedback	−0.06	(−0.4, 0.3)	0.7

**Table 5:** Mixed-effects generalized linear model of the glycemic profile change extracted from CGM data.

Glycemic Profile	β	95% CI	*p*
Average blood glucose			
**Visit Week**			
0	Ref		
4	2.7	(−4.3, 9.7)	0.4
8	3.3	(−3.6, 10.3)	0.3
12	3.3	(−7.9, 14.6)	0.6
**Intervention group**			
Control	Ref		
TLE + blinded CGM	−4.2	(−14.7, 6.1)	0.4
TLE + real-time feedback	−7.1	(−20.2, 6.0)	0.2
Estimated HbA1c			
**Week**			
0	Ref		
4	0.1	(−0.1, 0.3)	0.3
8	0.1	(−0.1, 0.3)	0.3
12	0.1	(−0.2, 0.4)	0.5
**Intervention group**			
Control	Ref		
TLE + blinded CGM	−0.2	(−0.5, 0.1)	0.2
TLE + real-time feedback	−0.3	(−0.7, 0.1)	0.2

## Data Availability

The datasets from this study will be available from the corresponding author on written request.
